# Meteorological Table

**Published:** 1811-05

**Authors:** 


### METEOROLOGICAL TABLE.

*From March 28, to April 26.*

| | D | Therm. | | | Barom. | | | Hygrom. dry | | | damp | | | Atmospherical Variations. |
|---|---|---|---|---|---|---|---|---|---|---|---|---|---|---|
| | 28 | 41 | 53 | 48 | $30^{4}$ | — | —$^{5}$ | 35 | 52 | 33 | | | | E .. F ... |
| | 29 | 46 | 55 | 47 | — | — | —$^{4}$ | 26 | 41 | 48 | | | | E .. SE .. F .... |
| | 30 | 43 | 55 | 51 | —$^{3}$ | —$^{2}$ | —$^{1}$ | 27 | 45 | 35 | | | | SE . C .... W . F .... N . |
| ◐ | 31 | 49 | — | 46 | —$^{1}$ | — | — | 25 | — | — | | | | E .. C .... R . |
| | 1 | 49 | 53 | 46 | 30 | $29^{9}$ | — | 26 | 33 | 24 | | | | SE. C .... F .... |
| | 2 | 47 | 58 | 53 | —$^{8}$ | — | — | 19 | 29 | 16 | | | | S .. SW .. F .. C .. F .... |
| | 3 | 49 | 58 | 56 | —$^{9}$ | — | 30 | 11 | 30 | 28 | | | | W .. NW . F ... — .... |
| | 4 | 52 | 60 | 52 | — | — | —$_{1}$ | 23 | 30 | 29 | | | | E .. SE .. F ... — .. |
| | 5 | 47 | 51 | 45 | —$^{1}$ | 30 | — | 22 | 30 | 25 | | | | E .. C ... F .. |
| | 6 | 40 | 59 | 49 | $29^{8}$ | —$^{7}$ | —$_{5}$ | 20 | 35 | — | | | | W .. C .... F .... R ... in n. |
| | 7 | 43 | 49 | 39 | — | — | — | 22 | — | — | — | 15 | 8 | NW .. R .. Snow . F .... |
| ○ | 8 | 38 | 44 | 39 | — | — | — | | | | 12 | 6 | 10 | E .. C .... F .. Snow . F ... |
| | 9 | 37 | 48 | 41 | —$^{6}$ | —$^{7}$ | — | — | 22 | 21 | 7 | — | — | NE ... F ... — .... — .... |
| | 10 | 37 | 49 | 48 | —$^{8}$ | — | — | 10 | 20 | — | — | — | 2 | N .. W . F .... R .. F .. |
| | 11 | 43 | 49 | 44 | —$^{9}$ | — | $30_{1}$ | — | — | 2 | 14 | 10 | — | E .. R . F .... — |
| | 12 | 49 | — | 47 | — | — | 30 | — | 10 | — | 14 | — | 5 | SW .. F .. C .. F . R . |
| | 13 | 49 | 59 | 55 | $29^{9}$ | — | 30 | 2 | — | — | — | 2 | 5 | SW .. R . C .. F .. |
| | 14 | 56 | 62 | 57 | —$^{1}$ | — | — | — | 6 | — | 10 | — | 5 | W .. F .. C . — .. |
| | 15 | 55 | 62 | 56 | — | — | 30 | | | | 10 | — | 10 | SW .. C .... F .... R .. in n. |
| ◑ | 16 | 55 | 57 | 52 | $29^{9}$ | —$^{8}$ | — | | | | 6 | — | 10 | SW .. C .... R ... — ... |
| | 17 | 48 | 57 | 55 | —$^{2}$ | — | —$_{3}$ | — | 20 | 40 | 6 | — | — | W . F .. — .... — ... |
| | 18 | 55 | 57 | 48 | —$^{8}$ | —$^{6}$ | —$_{4}$ | | | | 8 | 40 | — | E ... SW . W .. C .. F ... |
| | 19 | 48 | 55 | 50 | —$^{2}$ | — | —$_{3}$ | | | | 47 | — | 45 | E .. SW . F ... R ... F .... |
| | 20 | 52 | 56 | 54 | —$^{8}$ | —$^{4}$ | —$_{3}$ | | | | 47 | 45 | 48 | S .. F.. R ... — ... — .. in n. |
| | 21 | 58 | 61 | 56 | —$^{4}$ | —$^{5}$ | —$_{6}$ | | | | 60 | 27 | 30 | SW .. F ... — .... |
| | 22 | 55 | 65 | 60 | —$^{6}$ | — | —$_{5}$ | | | | 42 | 35 | 40 | SE . C .. R. F .... |
| ● | 23 | 60 | 70 | 63 | —$^{5}$ | —$^{7}$ | — | | | | 44 | 25 | 35 | E . R .. F . F .... — |
| | 24 | 59 | 68 | 65 | —$^{7}$ | —$^{8}$ | — | | | | 40 | 37 | 32 | SW . E .. F .... — |
| | 25 | 60 | 64 | 58 | —$^{7}$ | — | — | | | | 35 | 22 | 35 | E .. F .. C .. |
| | 26 | 57 | 64 | 55 | —$^{7}$ | —$^{6}$ | — | | | | 38 | 25 | 27 | E .. R .. F ... |

22. Lightning in the evening, from the West.
23. Lightning in the evening, from the South. On these days the heat oppressive, and the atmosphere highly electric.

Quantity of Rain from March 28 to April 26, at an elevation of 36 feet about the surface of Cavendish-Square, $\frac{3}{4}$ of an inch and $\frac{5}{100}$.

From the 23d of March to the 6th of April, the atmosphere was remarkably dry; from the 7th to the 17th of April, fluctuating between humidity and dryness; and from that day to the 26th, the hygrometer points a high state of humidity.

*Princes-Street, Cavendish-Square.*

